# Excellent Outcomes With the Selective Use of Oral Antibiotic Therapy for Bone and Joint Infections: A Single-Center Experience

**DOI:** 10.7759/cureus.26982

**Published:** 2022-07-18

**Authors:** Mason A Halouska, Zachary A Van Roy, Amanda N Lang, Jacey Hilbers, Angela L Hewlett, Nicolas W Cortes-Penfield

**Affiliations:** 1 Division of Infectious Diseases, University of Nebraska Medical Center, Omaha, USA

**Keywords:** application of oviva, oral antibiotic therapy, musculoskeletal infection, bone and joint infection, orthopedic infectious disease

## Abstract

Background and objective

Recent studies have challenged the notion that prolonged intravenous (IV) antibiotics are preferable to oral antibiotics for treating musculoskeletal infections. Our institution’s orthopedic surgery and orthopedic infectious disease (ID) groups have established consensus criteria for the use of oral antibiotics in musculoskeletal infections. In this study, we examine one-year and two-year outcomes of the selective use of oral antibiotics for musculoskeletal infections in a real-world setting.

Methods

We conducted a single-center retrospective analysis of adults seen in our orthopedic ID clinic over a six-month period for the first episode of surgically managed osteomyelitis, native joint septic arthritis (NJSA), prosthetic joint infection (PJI), or other musculoskeletal hardware infection with an established microbiologic etiology who received surgical interventions and >2 weeks of antimicrobial treatment. Patients were evaluated for treatment failure at one year and two years following their index surgery, which we defined as death, unplanned surgery, or the initiation of chronic antibiotic suppression.

Results

One-year treatment failure rates were 0/23 (0%) in patients who switched to oral therapy versus 6/17 (35%) in patients who remained on IV treatment. Two-year treatment failure rates were 0/23 (0%) in patients who switched to oral therapy versus 8/17 (47%) in patients who remained on IV treatment.

Conclusions

Our consensus criteria for the switch to oral antibiotics for musculoskeletal infections identified patients who went on to have excellent outcomes at one year and two years, suggesting that these criteria can effectively identify patients at low risk for treatment failure. Collaboration between ID specialists and orthopedic surgeons to select antimicrobial regimens can avoid significant burdens, costs, and complications associated with prolonged IV therapy.

## Introduction

Bone and joint infections lead to significant morbidity among adults, and the incidence of musculoskeletal infections is on the rise [1-3]. Traditionally, the treatment of bone and joint infections included prolonged intravenous (IV) antibiotic therapy; however, this practice does not have a strong evidentiary basis [4]. Moreover, prolonged IV therapy is associated with high costs, catheter-related complications, and economic and quality of life-related burdens for patients [5-8]. OVIVA, a recent randomized controlled trial, demonstrated the non-inferiority of oral (PO) versus IV antibiotic therapy for bone and joint infections [9].

The University of Nebraska Medical Center (UNMC) is a regional referral center for complex orthopedic infections. In 2019, our orthopedic infectious disease (ID) and orthopedic surgery groups met to review the data supporting the use of oral antibiotic therapy for musculoskeletal infections, which led to establishing consensus criteria for an early switch to oral antibiotics in these patients. In this study, we analyze one-year and two-year treatment outcomes at our center following the adoption of the consensus criteria.

## Materials and methods

Study design and subjects

We conducted a single-center retrospective analysis of patients receiving IV and oral antimicrobial therapy for musculoskeletal infections. We identified patients using an electronic medical record dataset including adults aged more than 18 years who had a clinic visit with one of two core orthopedic ID physicians (A.L.H. and N.W.C.) between July 1, 2019, and December 31, 2019, for osteomyelitis, native joint septic arthritis (NJSA), prosthetic joint infection (PJI), or other musculoskeletal hardware infections.

Eligible patients were those with an identified bacterial pathogen from surgical tissue, synovial fluid, or bone culture, no prior treatment failure or infection at that site, and treatment with surgery plus an oral or IV antimicrobial for more than two weeks. Notably, this allowed us to include patients treated with debridement, antibiotics, and implant retention (DAIR) in the study. We excluded patients initially planned (per clinical documentation during initial hospitalization from the treating ID physician) to receive indefinite antimicrobial suppression for retained infected orthopedic hardware, as well as patients with fungal, mycobacterial, or atypical bacterial organisms (e.g., Coxiella).

During this time period, we employed a switch to oral therapy for patients with musculoskeletal infections based on the following consensus criteria: (1) first episode of musculoskeletal infection at the affected anatomic site, (2) identification of a bacterial pathogen susceptible to one or more highly bioavailable antibiotics, (3) no concern from providers about patient’s ability to adhere to oral antibiotic therapy (principally based on recent prior no-shows to clinic appointments or patient-directed hospital discharges), (4) no vertebral infection, and (5) no concurrent infection necessitating IV therapy [e.g., complicated Staphylococcus aureus (S. aureus) bacteremia]. Following the creation of the consensus criteria, the two core orthopedic ID physicians were responsible for the implementation of the criteria for individual patient care. We obtained approval from UNMC’s Institutional Review Board (IRB) to collect and publish data for this study as exempt research.

Definitions

We classified PJI as early or late (i.e., arising <90 or >90 days from the original arthroplasty). We defined the index surgical procedure as the first surgery for treatment during the patient’s first hospital admission for a musculoskeletal infection. We classified index surgical procedures as irrigation and debridement (I&D) alone (including debridement and implant retention surgeries for PJI), I&D with hardware explantation and implantation of new hardware, I&D with hardware explantation and implantation of an antibiotic spacer, and I&D with hardware explantation and no implantation of new material. We recorded all antibiotics received for at least 72 hours after the index surgical procedure until the completion of therapy. We defined patients as being treated with oral antibiotics if they received any oral antibiotic therapy (other than rifampin) during their initial course of treatment, and as being treated with all-IV therapy if they received only IV therapy (other than rifampin) during their initial course of treatment. In considering the time before the switch to oral antibiotics, we classified an early switch to oral antibiotics as occurring within 14 days of the index surgery. For this study, the highly bioavailable antibiotics included fluoroquinolones, tetracyclines, trimethoprim-sulfamethoxazole, metronidazole, rifampin, linezolid, clindamycin, amoxicillin, and first-generation cephalosporins.

Variables and outcome measures

We collected data on patient demographics, comorbidities, anatomic site and type of infection, and orthopedic index surgical procedure with the subsequent antimicrobial course. For each patient, we identified the date and type of index surgical procedure and the organisms obtained from associated deep cultures. For each antibiotic administered for greater than 72 hours, we recorded the dose, the dates of administration, the reason for discontinuation, and any medication-related adverse effects. The primary outcome was treatment failure at one year from the index surgery, defined as a composite of death, unplanned surgery for the same infection, or unplanned initiation of chronic antibiotic suppression. We also assessed treatment failure at two years using the same composite definition.

Statistical analysis

We stratified patients based on the route of antibiotic administration (PO switch or all-IV). In addition to performing descriptive statistics, we compared categorical variables using the Chi-squared and Fischer’s exact tests and continuous variables using the student’s t-test, with a significance value of 0.05 used to assess statistically significant differences for all analyses. Analysis was performed and images were created using Graphpad Prism 9.0 (GraphPad Software, San Diego, CA).

## Results

Forty patients met the inclusion criteria, of whom 23 started or switched to oral antibiotics while 17 patients remained on IV antibiotics throughout their treatment. Table 1 displays the demographics and comorbidities of the cohort. Patients treated with oral antibiotics were younger and less likely to have chronic kidney disease; other characteristics were similar between groups.

**Table 1:** Patient demographics, comorbidities, and treatment outcomes *Statistically significant SD: standard deviation

	Patients who received IV antibiotics only (n=17)	Patients switched to PO antibiotics (n=23)	P-value
Demographics			
Age, years, mean (SD)	66.1 (16.9)	55.2 (16.2)	0.049*
Sex (female), n (%)	7 (41.2%)	7 (30.4%)	0.494
BMI, kg/m^2^, mean (SD)	33.3 (9.3)	30.3 (5.1%)	0.202
Comorbidities			
Diabetes mellitus, n (%)	5 (29.4%)	6 (26.1%)	0.822
Peripheral vascular disease, n (%)	2 (11.8%)	1 (4.3%)	0.392
Active tobacco use, n (%)	2 (11.8%)	1 (4.3%)	0.392
Chronic kidney disease, n (%)	5 (29.4%)	0 (0%)	0.005*
Rheumatoid arthritis, n (%)	0 (0%)	1 (4.3%)	0.397
Outcomes at one year			
Death, n (%)	0 (0%)	0 (0%)	N/A
Unplanned surgery at the same anatomic site, n (%)	3 (17.6%)	0 (0%)	0.069
Initiation of chronic antibiotic suppression, n (%)	5 (29.4%)	0 (0%)	0.009*
Composite treatment failure, n (%)	6 (35.3%)	0 (0%)	0.003*

**Figure 1:**
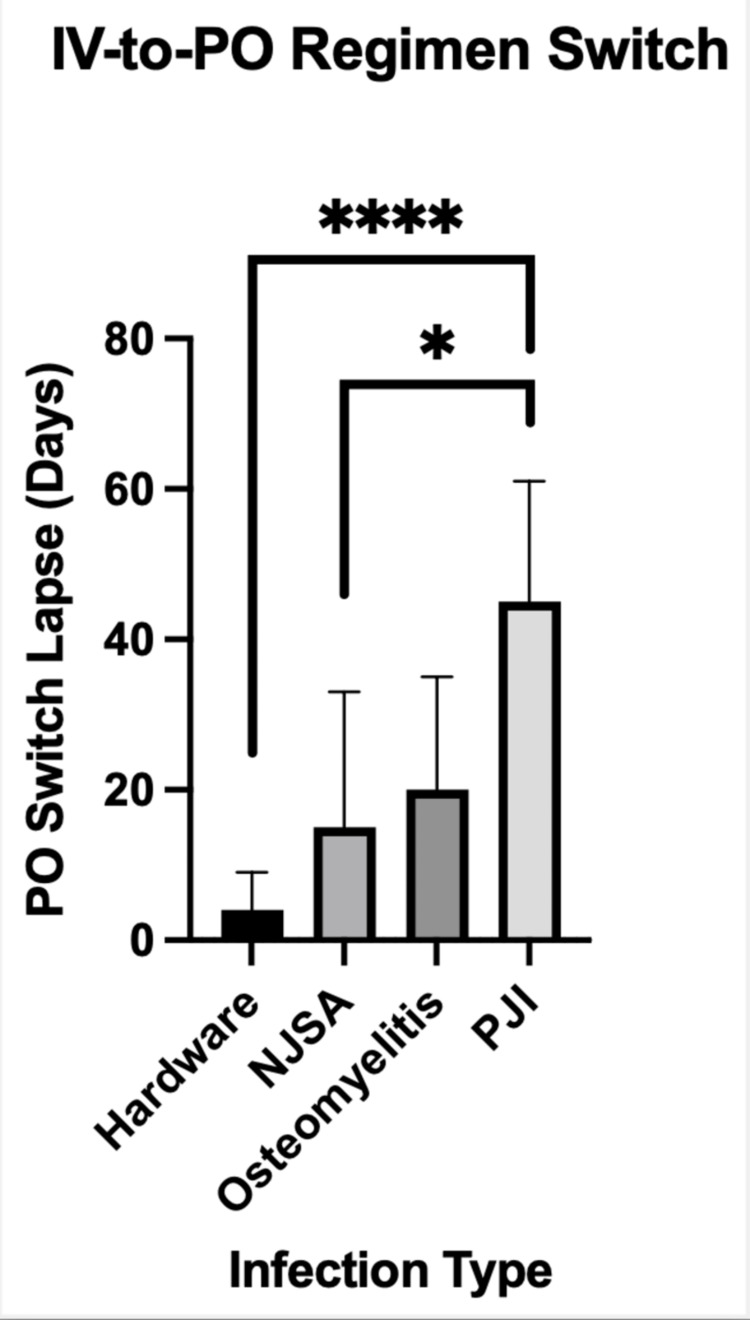
Duration between the initiation of IV antibiotics and the switch to oral antibiotics, according to infectious syndrome NJSA: native joint septic arthritis; PJI: prosthetic joint infection

The most common sites of infection were the knee (41.5%) and hip (22.0%). The most common infections were late PJI (37.5%), early PJI (17.5%), hardware infection other than PJI (17.5%), osteomyelitis (17.5%), and NJSA (10.0%). The microbiology of these infections included S. aureus (57.1% methicillin-susceptible) in 14 patients, coagulase-negative staphylococci in seven patients, streptococci in 12 patients, Enterobacteriaceae in eight patients, and other organisms in 10 patients. Eight patients had multiple organisms in cultures, though in five of these cases, the treating clinicians regarded one organism as the true pathogen and the others as likely contaminants. Figure 1 shows the means and standard deviations (SD) of the duration from the initiation of IV antibiotics to the switch to oral antibiotics based on the diagnosed infectious syndrome. Among all patients who switched to oral antibiotics, the mean period between index surgery and switch to oral antibiotics was 20.2 (SD: 18.1) days; however, when patients with PJI were excluded, the mean time to PO switch was just 10.1 (SD: 11.3) days. In total, one out of nine patients with PJI, two out of four patients with osteomyelitis, six out of six patients with other musculoskeletal hardware infections, and two out of four patients with NJSA had an early (within two weeks of index surgery) switch to oral antibiotics. In patients with PJI, six patients were treated with DAIR. In patients with PJI treated with DAIR, there was an average duration of 40 days before the switch to oral antibiotics.

We compared the one-year outcomes between patients in the oral antibiotic group and the IV antibiotic group (Table 1). No patients in either group died within one year of the index surgery. Patients in the oral antibiotic group had lower incidences of unplanned surgery at the same anatomic site (0% vs. 17.6%, p=0.037), unplanned initiation of chronic antibiotic suppression (0% vs. 29.4%, p=0.006), and overall treatment failure (0% vs. 35.3%, p=0.001) at one year versus those who received all-IV therapy. At two years of follow-up from the index surgery, outcomes for the oral and all-IV therapy groups were similar to those seen at one year, with a persistently lower incidence of treatment failure in the oral group (0% vs. 47.1%, p=0.0002). All the patients in the IV antibiotic group who experienced treatment failure at one or two years had a diagnosis of PJI, and these patients had an average age of 76 years.

The specific antibiotics and most common dosing regimen used for IV and oral therapy are shown in Table 2. There was no significant difference in the incidence of medication-related adverse events leading to a change in therapy between oral and intravenous antibiotic therapy (13.9% vs 18.8%, p=0.55). Adverse events with IV therapy included skin rash, hyperkalemia, acute kidney injury, thrombocytopenia, and leukopenia. Adverse events with oral therapy included skin rash, pruritis, fatigue, tremors, and pancytopenia.

**Table 2:** Intravenous and oral antibiotics: frequency of use and dosing regimen

	Number of patients	Most common dosing regimen
IV antibiotic		
Vancomycin	6	1 g daily
Cefazolin	3	2 g TID
Ceftriaxone	3	2 g daily
Ampicillin	2	12 g daily
Cefepime	2	2 g BID or 1 g daily
Daptomycin	1	1250 mg daily
Oral antibiotic		
Amoxicillin	8	1 g TID
Trimethoprim-sulfamethoxazole	7	800 mg–160 mg BID
Levofloxacin	3	750 mg daily
Amoxicillin/clavulanate	2	500 mg–125 mg TID
Cefadroxil	2	1 g BID
Cephalexin	1	1 g TID
Doxycycline	1	100 mg BID

## Discussion

In this retrospective analysis of one-year and two-year outcomes among patients with bone and joint infections, we noted no treatment failures in patients who switched to oral antibiotic therapy, versus a 35.3% rate of treatment failure at one year and a 47.1% rate of treatment failure at two years in patients who remained on IV antibiotics. Both the rates of unplanned antibiotic suppression and unplanned surgery were lower in the patients who switched to oral therapy versus those who were kept on IV therapy. Notably, the patients who experienced treatment failure in the IV antibiotic group had a higher average age of 76 years than the average age of 66 years for all patients treated exclusively with IV antibiotics. Our data suggest that our consensus criteria for the use of oral antibiotics in musculoskeletal infections can effectively identify patients at low risk for treatment failure. These criteria were primarily based on previously identified risk factors for treatment failure in PJI, and hence our findings are consistent with previously published literature. While we excluded patients with vertebral infections from switching to oral therapy, this was a temporary measure while we collected initial outcomes data given the potentially catastrophic consequences of treatment failure with vertebral infections; we do not have any compelling reason to believe that outcomes would differ with oral therapy in vertebral infection.

Our study adds more real-world data to support the idea that oral antimicrobials can be highly efficacious in the treatment of musculoskeletal infections. We did not directly consider the differences in economic and quality of life measures between oral and IV therapy, but previous studies have noted decreased economic and quality of life burdens with oral therapy in comparison to IV therapy. We have no reason to believe the same would not be true for our study population.

This study has a number of limitations. Most importantly, the sample size was small, and the study was retrospective, with selection bias resulting in patients predisposed to a poor outcome being kept on IV therapy. In this study, the selection bias resulted in older patients and patients with chronic kidney disease being less likely to transition to oral antimicrobials. Patients in this high-risk group may have fared similarly with oral antimicrobials as well. Therefore, we do not present these data as evidence that oral therapy is non-inferior to IV therapy (already demonstrated in the OVIVA trial), but rather that our low-risk criteria effectively identified patients who responded well to oral antimicrobial therapy. 

Secondly, only 11/23 patients in the oral antibiotic group started or switched to an oral antibiotic within two weeks of the index surgery. While we included osteomyelitis, NJSA, PJI, and other musculoskeletal hardware infections in this study because we believe that patients with all of these infections have the potential to benefit from an early transition to oral therapy, we also wanted to consider each infectious syndrome individually. In doing so, patients treated for PJI had the longest delay before switching to oral therapy. The delay in switching all patients with PJI to oral antibiotics in our study is likely due to several causes, including provider apprehension related to diverging from the established practice of IV antibiotics in PJI in the first months following the publication of OVIVA. However, as the duration of PJI therapy is frequently extended to 12 or even 24 weeks, even late transitions to oral antimicrobials have the potential to avoid economic and quality of life burdens associated with prolonged IV therapy. Even the patients with PJI treated with DAIR in our study likely experienced this benefit despite an average duration of 40 days before switching to oral antibiotics. Furthermore, most patients treated with oral antimicrobials in the OVIVA trial switched from initial IV therapy after about one week, and hence we consider the likelihood that earlier switches to oral therapy would have produced inferior outcomes in our cohort to be low.

## Conclusions

The findings of this study revealed that our use of consensus criteria to carefully select patients with musculoskeletal infections for treatment with oral antibiotics led to zero treatment failures at one year and two years. This suggests that our criteria are highly effective at identifying patients who are likely to do well with oral therapy and in whom the time burden, cost, and risks of IV antibiotic therapy should be avoided.
